# Supplementary material for: Causal Relationship between Aging and Anorexia Nervosa: A White-Matter-Microstructure-Mediated Mendelian Randomization Analysis
Source: Biomedicines. 2024 Aug 16;12(8):1874. doi: 10.3390/biomedicines12081874 (PMC11351342; doi:10.3390/biomedicines12081874)
Supplement: Supplementary file 1 [file biomedicines-12-01874-s001.zip › Supplementary Figure/Figure S4/Singe SNP plot of MR analysis for TL to ubm-b-1949.pdf]

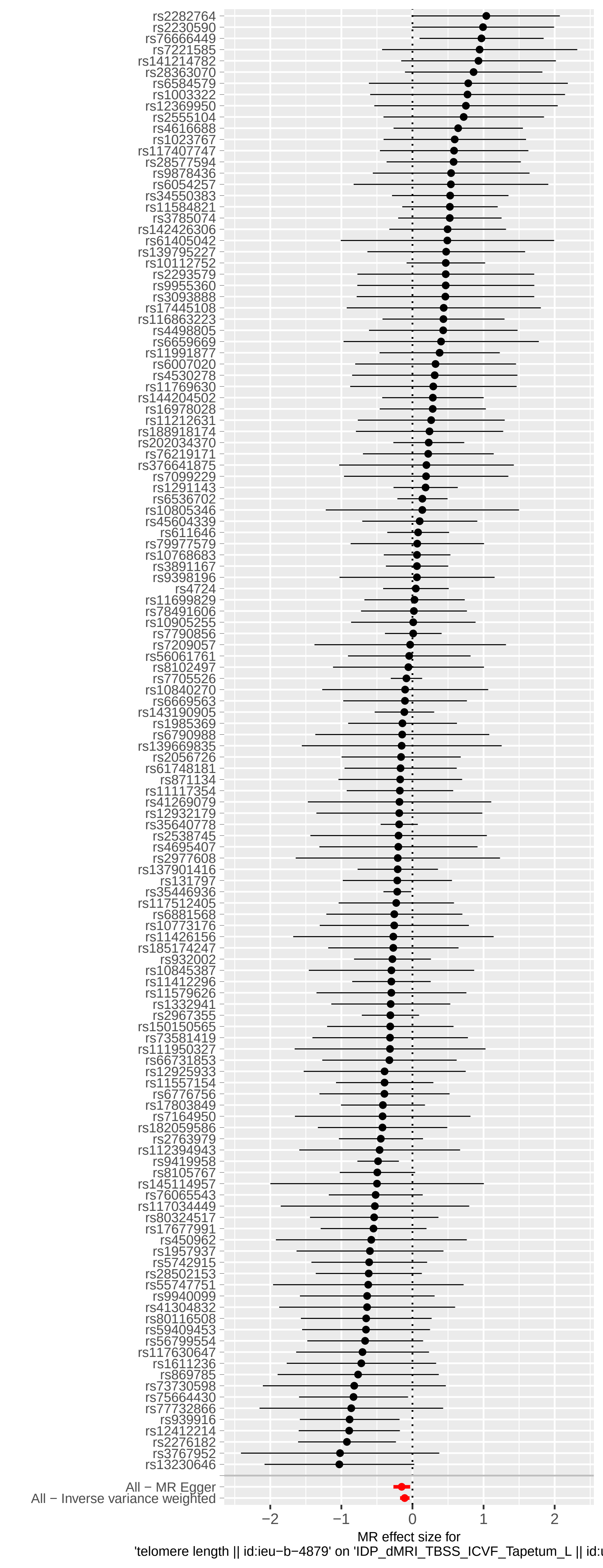

rs2282764
rs2230590
rs76666449
rs7221585
rs141214782
rs28363070
rs6584579
rs1003322
rs12369950
rs2555104
rs4616688
rs1023767
rs117407747
rs28577594
rs9878436
rs6054257
rs34550383
rs11584821
rs3785074
rs142426306
rs61405042
rs139795227
rs10112752
rs2293579
rs9955360
rs3093888
rs17445108
rs116863223
rs4498805
rs6659669
rs11991877
rs6007020
rs4530278
rs11769630
rs144204502
rs16978028
rs11212631
rs188918174
rs202034370
rs76219171
rs376641875
rs7099229
rs1291143
rs6536702
rs10805346
rs45604339
rs611646
rs79977579
rs10768683
rs3891167
rs9398196
rs4724
rs11699829
rs78491606
rs10905255
rs7790856
rs7209057
rs56061761
rs8102497
rs7705526
rs10840270
rs6669563
rs143190905
rs1985369
rs6790988
rs139669835
rs2056726
rs61748181
rs871134
rs11117354
rs41269079
rs12932179
rs35640778
rs2538745
rs4695407
rs2977608
rs137901416
rs131797
rs35446936
rs117512405
rs6881568
rs10773176
rs11426156
rs185174247
rs932002
rs10845387
rs11412296
rs11579626
rs1332941
rs2967355
rs150150565
rs73581419
rs111950327
rs66731853
rs12925933
rs11557154
rs6776756
rs17803849
rs7164950
rs182059586
rs2763979
rs112394943
rs9419958
rs8105767
rs145114957
rs76065543
rs117034449
rs80324517
rs17677991
rs450962
rs1957937
rs5742915
rs28502153
rs55747751
rs9940099
rs41304832
rs80116508
rs59409453
rs56799554
rs117630647
rs1611236
rs869785
rs73730598
rs75664430
rs77732866
rs939916
rs12412214
rs2276182
rs3767952
rs13230646
All − MR Egger
All − Inverse variance weighted
−2
−1
0
1
2
MR effect size for
'telomere length || id:ieu−b−4879' on 'IDP_dMRI_TBSS_ICVF_Tapetum_L || id:
